# P-1074. Clinical Outcomes in Patients Hospitalized with Exacerbation of Chronic Respiratory Infections treated with Ceftolozane/Tazobactam: Results from SPECTRA

**DOI:** 10.1093/ofid/ofae631.1262

**Published:** 2025-01-29

**Authors:** Emre Yucel, Alex Soriano, David Paterson, Florian Thalhammer, Stefan Kluge, Pierluigi Viale, Mike Allen, Brune Akrich, Yanbing Zhou, Huina Yang, Sundeep Kaul

**Affiliations:** Merck & Co., Inc., North Wales, Pennsylvania; Hospital Clínic de Barcelona, Barcelona, Catalonia, Spain; National University of Singapore, Singapore; Medizinische Universität Wien, Vienna, Wien, Austria; Department of Intensive Care, University Medical Center Hamburg-Eppendorf, Hamburg, Hamburg, Germany; Infectious Diseases Unit, Department of Medical and Surgical Sciences, Policlinico Sant'Orsola Malpighi, University of Bologna, Bologna, Italy, Bologna, Emilia-Romagna, Italy; MSD, UK, Ltd., London, England, United Kingdom; Merck Research Labs, MSD, Puteaux, Ile-de-France, France; Merck, Rahway, New Jersey; Tan Tock Seng Hospital, Singapore, Not Applicable, Singapore; Harefield hospital, london, England, United Kingdom

## Abstract

**Background:**

SPECTRA was a multicenter, observational study of 617 patients (pts) treated with ceftolozane/tazobactam (C/T), to inform the real-world clinical management of gram-negative infections.

**Table 1.**
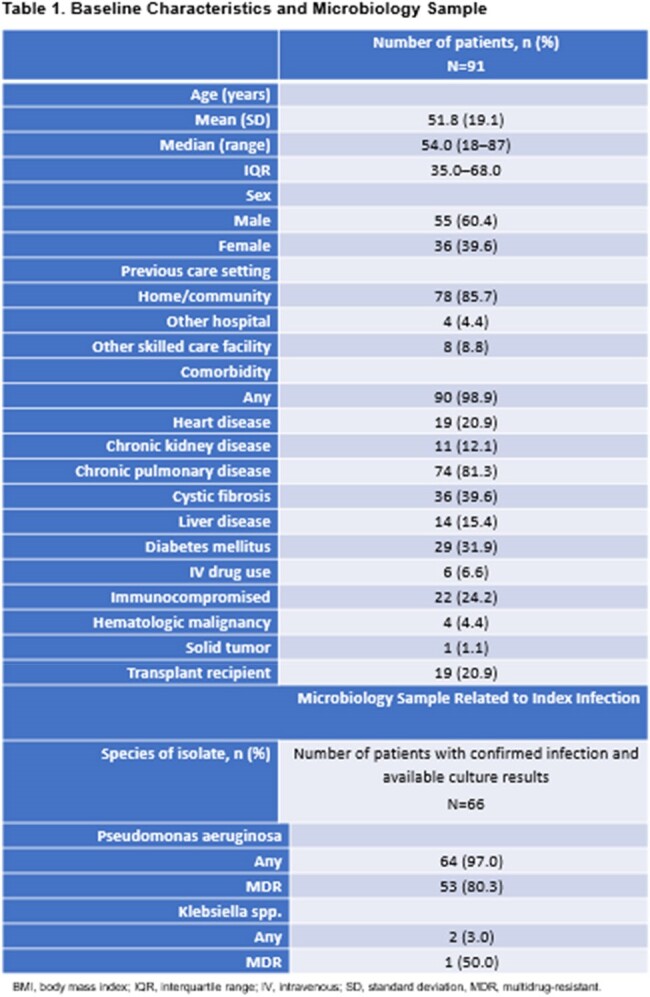
Baseline Characteristics and Microbiology Sample BMI, body mass index; IQR, interquartile range; IV, intravenous; SD, standard deviation, MDR, multidrug-resistant.

**Methods:**

Retrospective data for pts with an exacerbation of a chronic respiratory infection (CRI) were extracted from medical records < 6 months from the index date, < 30 days from the last dose of C/T, or until death as part of a subcategory analysis. Clinical success, all-cause in-hospital mortality (ACHM), mean length of stay (LOS) in intensive care unit (ICU), and 30-day all-cause re-admission were assessed.

**Table 2.**
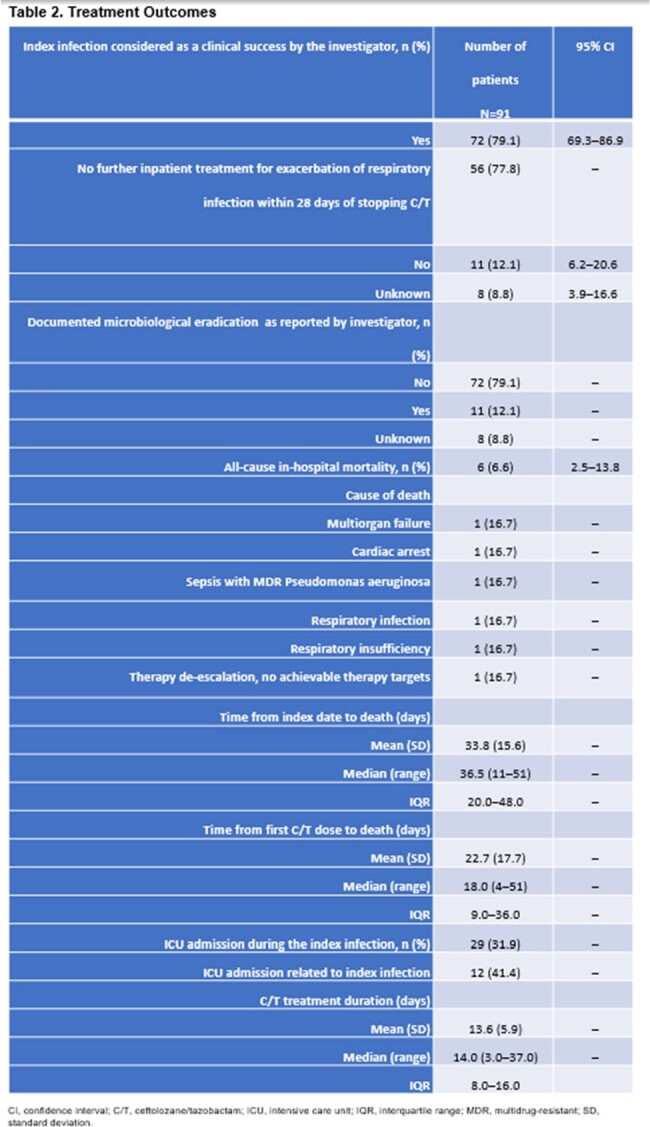
Treatment Outcomes CI, confidence interval; C/T, ceftolozane/tazobactam; ICU, intensive care unit; IQR, interquartile range; MDR, multidrug-resistant; SD, standard deviation.

**Results:**

Among 91 pts with CRI exacerbation, 98.9% (n=90/91) had ≥1 comorbidity (mean, 2.6; standard deviation [SD], 1.3), the most common of which was chronic pulmonary disease (81.3%, n=74/91; **Table 1**). Most pts with microbiology data had infection with multidrug-resistant *Pseudomonas aeruginosa* (80.3%, n=53/66; **Table 1**). Median C/T duration was 14.0 days (range: 3–37). Clinical success was seen in 79.1% of pts (n=72/91). Within 28 days of ending treatment, most pts (77.8%, n=56/72) did not require further treatment for the index infection, 81.9% (n=59/72) experienced resolution of CRI exacerbation, 12.1% (n=11/91) experienced microbiological eradication (**Table 2**). Median ICU LOS was 34.5 days (range: 3–58) in hospitalized pts (n=29). ACHM was 6.6% (n=6/91; 95% confidence interval [CI]: 2.5–13.8). The 30-day all-cause re-admission rate was 15.4% (n=14/91) and was infection-related in 7.7% of pts (n=7/91; **Table 2**).

**Conclusion:**

This sub-analysis of the SPECTRA study highlights the clinical characteristics and outcomes of pts hospitalized with CRI exacerbations treated with C/T. The sub-analysis shows positive clinical outcomes of C/T treatment, with high rates of clinical success and resolution of CRI exacerbation. Most patients did not require further antibacterial therapy within 28 days of stopping C/T, achieved clinical stability, and were discharged from the hospital, ICU, or step-down unit.

**Disclosures:**

**Emre Yucel, PhD**, Merck: I am a full time Merck Employee and own stocks in the retirement plan provided by Merck.|Merck: Stocks/Bonds (Public Company) **David Paterson**, bioMerieux: Grant/Research Support|bioMerieux: Honoraria|Merck: Advisor/Consultant|Merck: Grant/Research Support|Merck: Honoraria|Pfizer: Advisor/Consultant|Pfizer: Grant/Research Support|Pfizer: Honoraria|Shionogi: Grant/Research Support|Shionogi: Honoraria **Florian Thalhammer, MD**, MSD: Advisor/Consultant **Stefan Kluge, Prof. Dr. med.**, Merck & Co: Advisor/Consultant|Merck & Co: Board Member **Mike Allen, PhD**, Merck: I am a full time Merck Employee and own stocks in the retirement plan provided by Merck.|Merck: Stocks/Bonds (Public Company) **Brune Akrich, MD**, Merck: I am a full time Merck Employee and own stocks in the retirement plan provided by Merck.|Merck: Stocks/Bonds (Public Company) **Yanbing Zhou, PhD**, Merck: I am a full time Merck Employee and own stocks in the retirement plan provided by Merck.|Merck: Stocks/Bonds (Public Company)

